# Editorial: Self-compassion: From Neuroscience to Clinical Setting

**DOI:** 10.3389/fpsyg.2022.963738

**Published:** 2022-07-25

**Authors:** Andrea Poli, Angelo Gemignani, Christopher Chad Woodruff

**Affiliations:** ^1^Department of Clinical and Experimental Medicine, University of Pisa, Pisa, Italy; ^2^Department of Surgical, Medical and Molecular Pathology and of Critical Care Medicine, University of Pisa, Pisa, Italy; ^3^Department of Psychology, Northern Arizona University, Flagstaff, AZ, United States

**Keywords:** self-compassion, neuroscience (psychology), vagus nerve, PTSD, heart rate variability (HRV), self-reassurance

The expression “affective neuroscience,” which was first used in the early nineties of the twentieth century by Panksepp (2005), is now recognized as a distinct research field in brain science across species. Challenging the theory of constructed emotions (Feldman Barrett, 2016), Panksepp identified seven main emotional systems, including seeking, care, play, and lust on the positive side and fear, sadness, and anger on the negative side. The idea that imbalances in these ancient core emotional systems are significantly linked to psychiatric diseases like depression has been supported by an abundance of research into human clinical applications.

As a health-promoting affect (Sirois et al., 2015), compassion is defined as a deep awareness of others' suffering, followed by a desire to alleviate it and provide care, as well as understanding without judgment or pity (Gilbert, 2020). Self-compassion (SC) is compassion directed toward oneself in harsh or suffering situations. SC, unlike self-esteem, is independent of external factors and is linked to higher resilience and the ability to relate with oneself more tenderly (Conversano et al., 2020). Instead of feeling isolated or disconnected, SC allows the understanding of one's own flaws as part of the universal human experience and to see those experiences as part of human life (Neff, 2009; Poli et al., 2021). Previous research has shown that dispositional mindfulness, a construct that is closely related to compassion and SC, was associated with less self-reported emotional distress in many different populations (Hashizume et al., 2021; Poli et al., 2022). However, previous research has also suggested that dispositional SC, rather than dispositional mindfulness, may be an even more effective buffer against emotional distress. In fact, preliminary results from a pilot study indicated that dispositional SC is a stronger predictor of vagally-mediated heart rate variability (vmHRV) than mindfulness (Svendsen et al., 2020). Accordingly, the findings are in line with recent trends in the clinical mindfulness field (Feldman and Kuyken, 2011; Segal et al., 2018; Conversano et al., 2020), which point to a greater explicit focus on SC in mindfulness-based interventions and support clinical work targeting SC (Conversano et al., 2020). This Research Topic collects six articles developing our understanding of how SC interventions are able to improve psychophysiological responses and psychological wellbeing in clinical and non-clinical populations, or of what might be the effects of a trauma-informed mindful recovery program on SC.

Woodfin et al. carried out a randomized wait-list control trial, pre-registered at Clinicaltrials.gov (ID: NCT03453437), that investigated the effects of a 3-week intervention consisting of four seminars and a silent half-day retreat with short lectures and relevant experiential practices from Mindful Self-Compassion (MSC) and Mindfulness Based Stress Reduction (MBSR) on SC, maladaptive perfectionism, anxiety, depression, and body image. University students were randomly assigned to the intervention group and wait-list control group, and filled out self-report measures weekly, pre- and post-treatment. Eighty-nine participants completed the intervention and in the intervention group, when compared to the wait-list group, ANOVA revealed significant post-intervention reductions in maladaptive perfectionistic tendencies, depression and anxiety symptoms, as well as increased SC and improved body image. The authors found that mixed level modeling revealed statistically significant changes in SC, maladaptive perfectionism, adaptive perfectionism, anxiety, and depression but not body image.

Svendsen et al. investigated how SC was related to vmHRV in patients with recurrent major depressive disorder (MDD). In particular, the authors hypothesized that higher SC would associate with lower ruminative tendencies and with higher vmHRV in adults with recurrent MDD. Sixty-three participants with a history of at least three, or more, episodes of major depression filled out self-report measures of SC, depression and rumination. ECG was acquired while resting and the square root of the mean squared differences of successive RR interval values (RMSSD) was calculated as measure of vmHRV. Results confirmed the first hypothesis that higher SC would be associated with lower ruminative tendencies in recurrent MDD. However, the authors did not find that the tendency to be more self-compassionate was associated with higher vmHRV, while higher age predicted lower vmHRV across all statistical analyses.

Fifty-six armed forces veterans were studied by Gerdes et al. and listened to a loving-kindness meditation for SC (LKM-S), and had their heart rate, skin conductance, and HRV recorded throughout. Participants completed state measures of hyperarousal and social connectedness before and after the LKM-S, and PTSD symptom severity, dispositional emotion regulation, and SC were also examined. Overall, the findings showed that a one-off compassion meditation exerted transitory benefits on self-report measures of SC and hyperarousal, but not on physiological responses. The results further imply that a SC-based strategy may be acceptable for veterans suffering from emotional distress, such as PTSD, but they also emphasize the importance of individual differences and the necessity for a longer-term intervention in these populations.

Creaser et al. investigated how individuals with full PTSD, those without PTSD, and those with subclinical PTSD responded to a validated SC exercise. The authors used electroencephalography (EEG) alpha-asymmetry and EEG microstate analysis to characterize brain activity time series during the SC exercise in the three groups, as well as heart rate, skin conductance, and HRV. In addition, self-reported changes in state mood and self-perception were assessed. During the SC exercise, directing SC toward oneself activated the negative self and produced a threat response in all three groups, with individuals with subclinical PTSD and high levels of hyperarousal having the most elevated threat response. Furthermore, the three PTSD groups differed in their activation of the EEG microstate related with saliency, attention, and self-referential processing brain networks. The authors argue that these results may provide potential neural biomarkers for quantitatively differentiating PTSD subgroups.

Moore et al. carried out a pilot study with 18 individuals investigating the effects of the Mindful Recovery Opioid use disorder Care Continuum (M-ROCC), during a buprenorphine office-based opioid treatment (OBOT), on the development of SC. In addition, differences in SC during the intervention among participants with diverse levels of trauma exposure, as indicated by high levels of childhood adversity, were investigated. The researchers concluded that M-ROCC may improve SC in OUD patients during OBOT by enhancing compassionate self-responding and lowering uncompassionate self-responding. Patients with OUD who had experienced more childhood adversity had lower levels of SC, which M-ROCC increased.

Using functional magnetic resonance imaging on 40 individuals, Kim et al. investigated how self-reassurance, defined as a compassionately-motivated cognitive self-relating style, may regulate negative emotion, after that participants were invited to engage in self-criticism and self-reassurance toward written descriptions of negative life events. Results, showed that neural markers of negative emotion are down-regulated during attempts to be compassionate and reassuring to one's suffering.

In light of the findings of this Research Topic, SC interventions may be beneficial for a variety of clinical and non-clinical populations and may represent a non-invasive target to promote psychological and physiological well-being, as well as a possible prevention strategy to preserve mental and physiological health.

## Author contributions

Conceptualization and writing—original draft preparation: AP. Validation, resources, and project administration: AP and CW. Writing—review and editing: AP, AG, and CW. Supervision: AG. All authors have read and agreed to the published version of the manuscript.

## Conflict of interest

The authors declare that the research was conducted in the absence of any commercial or financial relationships that could be construed as a potential conflict of interest.

## Publisher's note

All claims expressed in this article are solely those of the authors and do not necessarily represent those of their affiliated organizations, or those of the publisher, the editors and the reviewers. Any product that may be evaluated in this article, or claim that may be made by its manufacturer, is not guaranteed or endorsed by the publisher.
